# A dock and coalesce mechanism driven by hydrophobic interactions governs Cdc42 binding with its effector protein ACK

**DOI:** 10.1074/jbc.M117.789883

**Published:** 2017-05-24

**Authors:** George J. N. Tetley, Helen R. Mott, R. Neil Cooley, Darerca Owen

**Affiliations:** From the ‡ Department of Biochemistry, University of Cambridge, 80 Tennis Court Road, Cambridge CB2 1GA, United Kingdom and; § Isogenica Ltd., Chesterford Research Park, Little Chesterford, Essex CB10 1XL, United Kingdom

**Keywords:** CDC42, cell signaling, intrinsically disordered protein, protein conformation, protein motif, protein structure, protein-protein interaction, small GTPase, tyrosine-protein kinase (tyrosine kinase), samll

## Abstract

Cdc42 is a Rho-family small G protein that has been widely studied for its role in controlling the actin cytoskeleton and plays a part in several potentially oncogenic signaling networks. Similar to most other small G proteins, Cdc42 binds to many downstream effector proteins to elicit its cellular effects. These effector proteins all engage the same face of Cdc42, the conformation of which is governed by the activation state of the G protein. Previously, the importance of individual residues in conferring binding affinity has been explored for residues within Cdc42 for three of its Cdc42/Rac interactive binding (CRIB) effectors, activated Cdc42 kinase (ACK), p21-activated kinase (PAK), and Wiskott–Aldrich syndrome protein (WASP). Here, in a complementary study, we have used our structure of Cdc42 bound to ACK via an intrinsically disordered ACK region to guide an analysis of the Cdc42 interface on ACK, creating a panel of mutant proteins with which we can now describe the complete energetic landscape of the Cdc42-binding site on ACK. Our data suggest that the binding affinity of ACK relies on several conserved residues that are critical for stabilizing the quaternary structure. These residues are centered on the CRIB region, with the complete binding region anchored at each end by hydrophobic interactions. These findings suggest that ACK adopts a dock and coalesce binding mechanism with Cdc42. In contrast to other CRIB-family effectors and indeed other intrinsically disordered proteins, hydrophobic residues likely drive Cdc42–ACK binding.

## Introduction

Cdc42 is a small G protein of the Rho family, which has a well-established role in regulating the actin cytoskeleton, being particularly associated with the formation of filopodia (reviewed in Ref. 1). By regulating the cellular architecture, Cdc42 also plays roles in cell motility, survival, and proliferation. Although the introduction of a Cdc42 mutant into fibroblasts showed hallmarks of transformation, activating mutations of Cdc42 have not been found in human cancers (2). Gene targeting, however, has demonstrated an essential role for Cdc42 downstream of Ras (3). As such, it represents a potential route to inhibit oncogenic signaling pathways driven by aberrant Ras activation.

The paradigm of G protein activation describes binding of GTP, which results in an alteration in the structure of the G proteins when the γ-phosphate of GTP forms hydrogen bonds with residues in two flexible surface regions, switches I and II, thus constraining them in a more rigid conformation that is competent to bind effector proteins. The switch regions of Ha-Ras have been demonstrated to adopt two different conformational states even in the GTP-bound, active form. State 1 is analogous to the GDP-bound form, whereas state 2 is the conformation competent to engage effector proteins (4). For Ha-Ras, state 2 is itself stabilized by effector binding and is sufficiently populated to allow signal propagation. In contrast, Cdc42 exhibits a far greater degree of flexibility even in the active form, and crystal structures of Cdc42·GDP and Cdc42·GMPPCP along with phosphorus NMR studies suggest that the active and inactive forms of Cdc42 are conformationally similar. The presence of an effector protein shifts the equilibrium of Cdc42·GMPPCP^3^ and allows it to adopt the active conformation with rigidified switch regions (5).

Like many small G proteins, active Cdc42 interacts with a large number of downstream effector proteins. One family of these effectors is defined by a consensus sequence known as the Cdc42/Rac interactive binding (CRIB) motif (6), contained within their G protein–binding region (GBD). Several structures are available describing Cdc42 in complex with the GBDs from CRIB family effector proteins (7). In each, the GBD is seen to wrap around the G protein, forming an intermolecular β-sheet and interacting with both the switch I and II regions (8–10). A comparison of all of the available structures shows that the CRIB consensus regions bind similarly to Cdc42, with specificity determinants and major structural differences lying outside this motif in the remainder of the GBD sequences. Activated Cdc42-associated kinase (ACK) is one such CRIB effector. ACK binds Cdc42 with nanomolar affinity (8) and is, moreover, specific for Cdc42 to the exclusion of closely related Rho-family G proteins, including Rac1 and TC10 (11, 12). ACK is a non-receptor tyrosine kinase, which functions downstream of growth factor receptors and has been implicated in survival, neuronal signaling, and androgen receptor activation through identification of its phosphorylation targets. ACK has also been implicated as an important driver of tumorigenesis and therefore is itself a potential therapeutic target (reviewed in Ref. 13).

Interestingly, the Cdc42-binding region of ACK (GBD) has the ability to reverse cellular transformation caused by expression of v-Ha-Ras in NIH3T3 fibroblasts (14). Promisingly, fusion of a cell-penetrating sequence to the ACK GBD resulted in a peptide that could enter cells and still retained the ability to inhibit Ras-driven phenotypes (14). Improvement of the affinity and druglike properties of this Cdc42-binding sequence could therefore generate a specific, competitive inhibitor for Cdc42 effector signaling with potential as a clinically useful inhibitor for Ras-driven cancers. With this in mind, data pertaining to the thermodynamics and mechanism of binding of the ACK GBD to Cdc42 would be particularly useful to inform inhibitor design.

**Figure 1.**
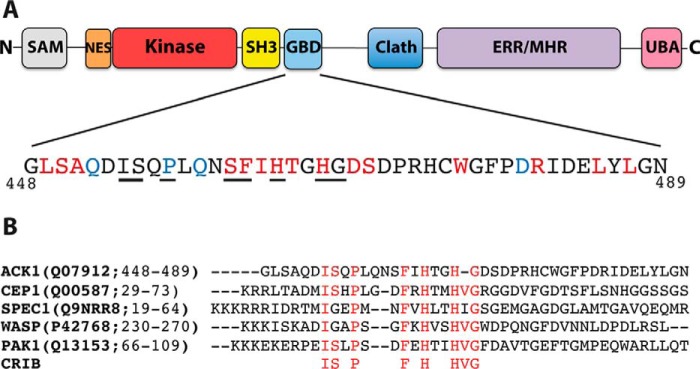
*A*, domain architecture of ACK. Structural domains and regions with assigned functions are highlighted: sterile α motif domain (*SAM*), nuclear export signal (*NES*), tyrosine kinase domain (*Kinase*), Src homology 3 domain (*SH3*), GBD, clathrin-interacting region (*Clath*), EGFR-binding region/Mig6 homology region (*ERR/MHR*), and ubiquitin-associated domain (*UBA*). The GBD is expanded to show the sequence *below* and the selection of residues in the ACK GBD that were selected for mutagenesis on the basis that they were most likely to be important in Cdc42 binding. Residues *colored red* were identified by their proximity to hot spot Cdc42 residues discovered in our previous studies (8, 20). Residues *colored blue* were identified by computational predictions from a selection of programs as described under “Results.” *Underlined residues* are part of the conserved CRIB motif. *B*, sequence alignment of ACK with other Cdc42/Rac-interacting proteins containing the CRIB motif. The residues and accession numbers for the Cdc42-binding region of each protein are shown. The CRIB consensus motif is shown *below* the alignment. Residues in each protein that conform to the consensus are *highlighted* in *red*.

ACK, like many signaling proteins, has a modular domain architecture and contains several identifiable structured domains (Fig. 1*A*). The GBD encompasses residues 448–489 (8) and is intrinsically disordered, showing no discernable secondary structure when free in solution and only a short segment of β-strand even when complexed with Cdc42 (8). Intrinsically disordered proteins (IDPs) or proteins containing intrinsically disordered regions (IDRs) like ACK often function in signaling pathways, with their disordered segments imparting a superior level of control and regulation of the interactions they mediate (15). The mechanism by which these proteins interact with their target partners is referred to as coupled folding and binding. This has been refined into a sequential model termed “dock and coalesce.” Here, binding is initiated by one segment of the IDR, which docks with a subsite on the partner protein interface. This is rapidly followed by the coalesce phase, where the remaining portions of the IDR bind to their cognate ancillary sites (16, 17).

Although we have structural information on the complex, there are few details on the mechanism of binding of ACK to Cdc42. This system is particularly interesting because not only is the ACK GBD disordered, but to some extent so is its target binding site, because it involves the flexible switch regions of Cdc42. The mechanism of binding to Cdc42 has been explored for another member of the CRIB family of proteins, the Wiskott–Aldrich syndrome protein (WASP) (18). In WASP, the docking region has been identified as the basic region (BR) at the N terminus of the GBD. Binding of the BR is the rate-limiting step for complex formation, and coalescence occurs very rapidly following docking (18). In fact, the importance of the BR has been well documented because it underpins the electrostatic steering mechanism utilized by WASP to contact Cdc42 (19). A similar mechanism has been postulated for PAK1, another member of the CRIB effector family (18). Furthermore, for most of the systems studied involving IDPs, long-range charge–charge interactions drive initial complex formation (17).

Previously, we have solved the structure of Cdc42 bound to the ACK GBD (8). The conformation adopted by ACK in complex with Cdc42 is significantly different from those of other CRIB effector proteins. All of the CRIB proteins form a short section of intermolecular β-sheet with Cdc42. In addition, WASP and PAK (PAK1 and -6) also form a β-hairpin and short α-helix toward the C terminus of their GBD, whereas ACK is otherwise unstructured. We have also undertaken thermodynamic studies to identify the residues in the binding interface on Cdc42 that contribute energetically to complex formation (20) and the residues that allow ACK to discriminate between Cdc42 and the closely related Rho family protein Rac1 (21). These studies pinpointed several residues in ACK that are likely to interact with the key contributors to binding energy on Cdc42. For instance, Val-42^Cdc42^ and Leu-174^Cdc42^ have been identified as contributing a large fraction of the binding energy between ACK and Cdc42, and these pack against Ile-463 and Leu-449 in ACK, whereas the D38A mutant of Cdc42 results in a 67-fold decrease in affinity and likely interacts with His-464 and His-467 of ACK in the complex structure.

Although a few individual residues in ACK have been mutated in previous studies, to our knowledge, a comprehensive screen has never been undertaken to dissect the energetic landscape of the ACK GBD in complex with Cdc42. To obtain the necessary data to direct a rational approach to using the ACK GBD as the basis for an experimental or therapeutic tool to inhibit Cdc42/Ras signaling and to obtain some mechanistic insight into the ACK–Cdc42 binding process, we have undertaken a thorough thermodynamic study on the ACK GBD. A panel of residues in ACK was mutated to interrogate the energetic environment of the Cdc42–ACK interaction. The data reveal the full thermal map of the ACK binding surface and indicate the critical input of conserved CRIB residues to stabilizing the complex. In contrast to WASP and PAK, and indeed several other IDP–target complexes, the initial encounter complex of Cdc42 and ACK is unlikely to be driven by electrostatic interaction but rather by hydrophobic interactions at the N terminus of the region.

## Results

### Residue selection

The residues selected for mutation are shown in Fig. 1*A*. First, we identified likely residues in ACK that were packed closest to the Cdc42 hot spots (residues whose mutation reduced ACK binding affinity > 10-fold), using the Cdc42–ACK complex structure (PDB code 1CF4) (8) and thermodynamic data (20). The same structure was used to inform computational predictions of the contribution of specific residues using mCSM (22), Robetta (23), and BeAtMuSiC (24). The outputs from the programs were quite different, so only those residues that produced a predicted shift in ΔΔ*G* that was in the top quartile in at least two programs were selected. Most residues identified had already been selected, but four additional residues were included: Gln-452, Pro-457, Gln-459, and Asp-480. Residues were also included if they were conserved across the consensus regions of CRIB family proteins (Fig. 1*B*), which expanded the selection to include Ile-454 and Ser-455. Finally, Leu-485 and Leu-487 were also mutated because they had been identified in the literature as potentially important in the Cdc42–ACK interaction (25). All residues were changed to alanine to allow calculation of the energetic contribution of their side chains to the complex (26). Ala-451 was substituted with aspartate to alter the hydrophobic character of this region and potentially introduce steric clashes with Glu-178^Cdc42^.

### Binding affinity analysis

**Figure 2.**
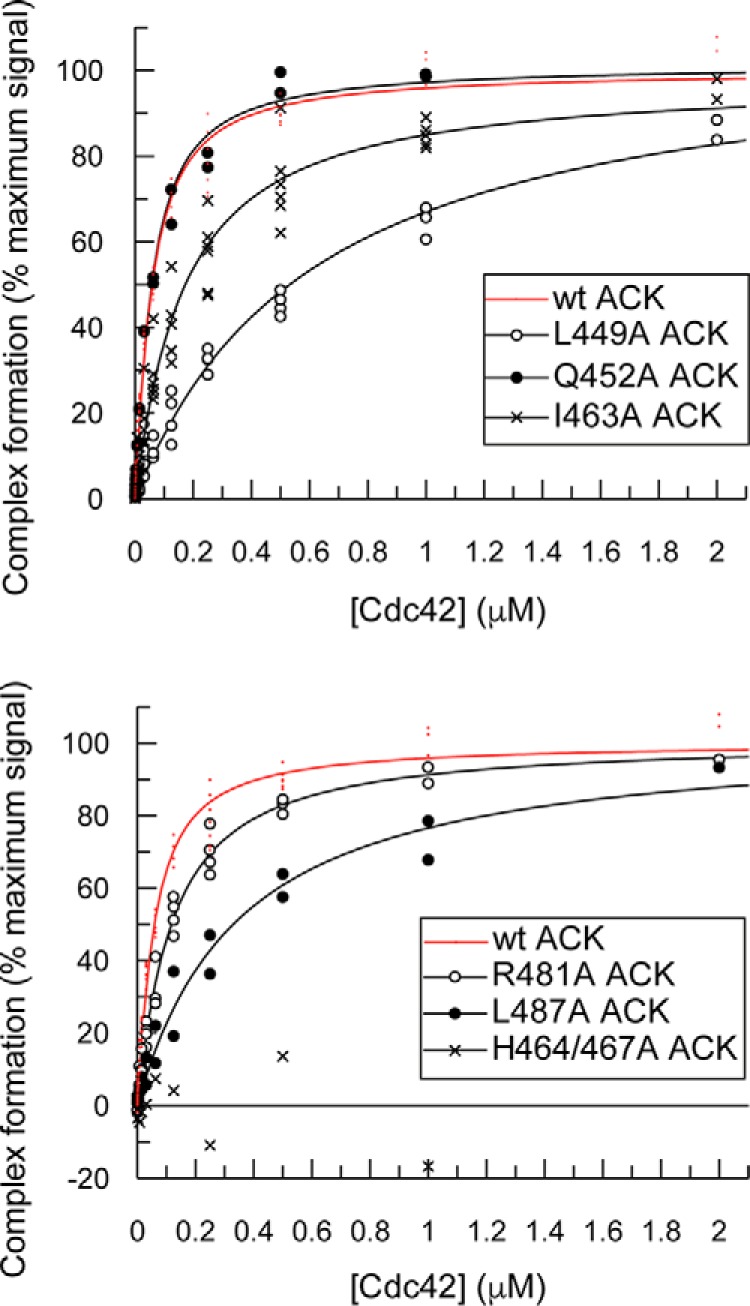
**Direct SPA binding data for the ACK GBD and mutant variants with Cdc42.** The indicated concentration of [^3^H]GTP-labeled Cdc42 was incubated with GST-tagged ACK GBD variants, as appropriate, in each SPA. The SPA signal was corrected by subtraction of the background signal from parallel measurements in which the effector protein was omitted. The effect of the concentration of Cdc42 on this corrected SPA signal was fitted to a binding isotherm to give an apparent *K_d_* value and the signal at saturating Cdc42 concentrations. The data and curve fits are displayed as a percentage of this maximal signal.

The apparent *K_d_* values for the interaction between the ACK mutants and Cdc42 were determined by direct scintillation proximity assays (SPAs). Example binding isotherms are shown in Fig. 2 and the affinities are summarized in Table 1.

**Table 1:** **Equilibrium binding constants and thermodynamic data for ACK GBD mutants**

Mutation	*K_d_**^a^*	Increase	Δ*G**^b^*	ΔΔ*G*
	*nm*	*-fold*	*cal/mol*	*cal/mol*
WT	44.2 ± 3.2		−10,027	-
L449A	491.5 ± 3.7	11.1	−8601	1426
S450A	167.1 ± 19.4	3.8	−9240	787
A451D	189.1 ± 28.8	4.3	NA*^c^*	NA
Q452A	44.0 ± 4.5			
I454A	ND*^d^*	>22.6*^e^*	>8181	>1846
S455A	243.1 ± 42.5	5.5	−9018	1009
P457A	ND	>22.6	>8181	>1846
Q459A	29.8 ± 5.4			
S461A	ND	>22.6	>8181	>1846
F462A	ND	>22.6	>8181	>1846
I463A	151.7 ± 13.8	3.4	−9297	730
H464A	ND	>22.6	>8181	>1846
T465A	85.4 ± 10.3	1.9	−9637	390
H467A	ND	>22.6	>8181	>1846
G468A	67.3 ± 19.0	1.5	−9778	249
D469A	38.0 ± 4.9			
S470A	35.4 ± 5.5			
W476A	137.0 ± 24.4	3.1	−9358	669
D480A	164.2 ± 30.9	3.7	−9250	777
R481A	107.4 ± 10.7	2.4	−9502	525
L485A	31.4 ± 3.4			
L487A	330.3 ± 54.6	7.5	−8837	1190
H464A/H467A	ND	>22.6	>8181	>1846
Δ17	ND	>22.6	>8181	>1846

*^a^* S.E. from curve fitting.

*^b^* Calculated using Δ*G* = *RT* ln*K_d_*^−1^, where *R* = 1.987 cal K^−1^ mol^−1^ and *T* = 298 K.

*^c^* NA, mutation to Asp does not allow Δ*G* calculation.

*^d^* ND, not determined (no binding); data that could not be fitted to the binding isotherm.

*^e^* Assuming a *K_d_* of > 1 μm (the limit of accurate direct SPA calculations).

**Figure 3.**
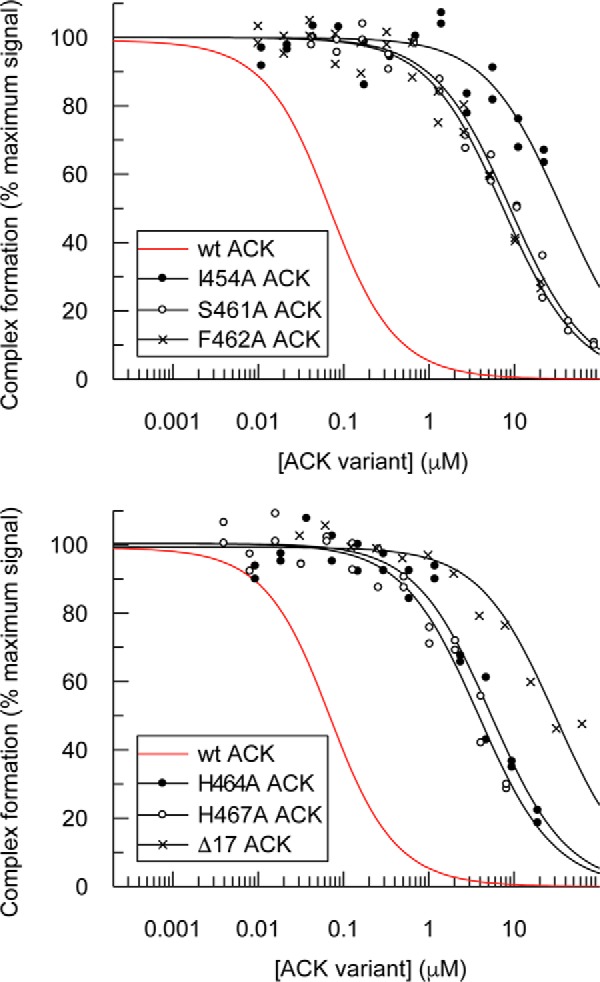
**Displacement of [^3^H]GTP·Cdc42 from GST–WT ACK GBD by ACK GBD variants.** Increasing concentrations of ACK GBD variants were titrated into fixed concentrations of [^3^H]GTP·Cdc42 and GST–WT ACK GBD in competition SPAs. The *K_d_* for Cdc42 binding to GST–WT ACK GBD was fixed to the values obtained for this *K_d_* in direct SPAs. The data were fitted to an isotherm describing a pure competition model to give an apparent *K_d_* (*K_i_*) value for the ACK variants. The data and curve fits are displayed as a percentage of the maximal SPA signal in each assay.

Of the 22 individual residues mutated, four (L449A, A451D, S455A, and L487A) caused disruption of binding significantly enough to raise the *K_d_* at least 4-fold in direct binding experiments. Six further mutations (I454A, P457A, S461A, F462A, H464A, and H467A) destabilized the final complex so profoundly that binding curves could not be obtained in these assays, because data gathered at higher concentrations (> 2 μm to achieve maximal binding) suffered from inaccuracy due to nonspecific binding effects. Competition SPAs were therefore performed, by titrating the mutant ACK peptides into a preformed complex of wild-type ACK and Cdc42, to obtain more accurate quantitative data by employing higher concentrations of the relevant ACK mutant. Selected binding isotherms are shown in Fig. 3, and the affinities are summarized in Table 2. These data accurately quantify the binding affinities for these deleterious mutations, showing that this subset of mutations have a large effect on *K_d_*, with decreases in affinity ranging from ∼35- to ∼1000-fold.

**Table 2:** **Equilibrium binding constants and thermodynamic data for ACK GBD mutants calculated from competition binding SPAs**

Mutation	*K_d_**^a^*	Increase	Δ*G**^b^*	ΔΔ*G*
	*nm*	*-fold*	cal/mol	cal/mol
WT	20 ± 5		−10,497	
I454A	19,730 ± 2340	986.5	−6415	4082
S455A	750 ± 160	37.5	−8351	2146
P457A	3830 ± 500	191.5	−7385	3112
S461A	4160 ± 570	208.0	−7336	3161
F462A	3460 ± 700	173.0	−7446	3051
H464A	2510 ± 290	125.5	−7636	2861
H467A	1790 ± 170	89.5	−7836	2661
H464A/H467A	ND*^c^*			
Δ17	4760 ± 110	238.0	−7257	3240

*^a^* S.E. from curve fitting.

*^b^* Calculated using Δ*G* = *RT* ln*K_d_*^−1^, where *R* = 1.987 cal K^−1^ mol^−1^ and *T* = 298 K.

*^c^* ND, not determined (no binding); data that could not be fitted to the binding isotherm.

### Interactions involving the conserved residues of the CRIB motif

Because the CRIB motif has been identified as a common and conserved motif in Cdc42 and Rac1 effectors (6), it would be expected to contain residues important for binding. The CRIB consensus has been defined as IS*X*P*XXXX*F*X*H*XX*HVG, although several proteins do differ slightly; for instance, in ACK, the penultimate valine is not present (Fig. 1*B*). Our data demonstrate that several conserved residues of the CRIB region are crucial in maintaining ACK binding affinity for Cdc42 and quantify their contribution.

**Figure 4.**
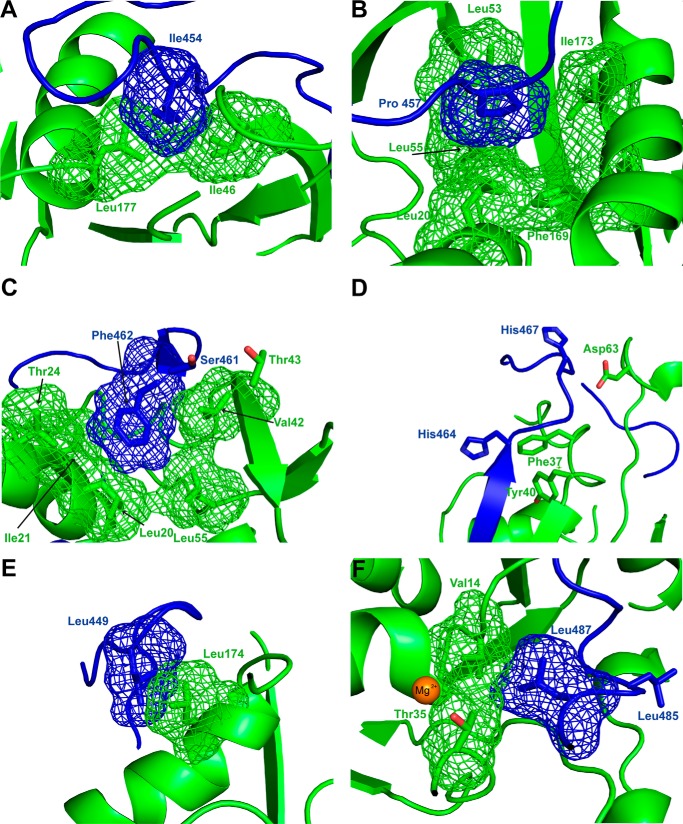
*A*, *schematic representation* of part of the Cdc42–ACK GBD structure (8) showing the contacts made by Ile-454^ACK^. The van der Waals surfaces of relevant residues are shown as a *mesh*. Cdc42 is *colored green*, and ACK is *colored blue. B*, *schematic representation* of part of the Cdc42–ACK GBD structure showing the contacts made by Pro-457^ACK^. *Coloring* is as in *A. C*, *schematic representation* of part of the Cdc42–ACK GBD structure showing the contacts made by Ser-461^ACK^ and Phe-462^ACK^. *Coloring* is as in *A. D*, *schematic representation* of part of the Cdc42–ACK GBD structure showing the contacts made by His-464^ACK^ and His-467^ACK^. *Coloring* is as in *A. E*, *schematic representation* of part of the Cdc42–ACK GBD structure showing the contacts made by Leu-449^ACK^. *Coloring* is as in *A. F*, *schematic representation* of part of the Cdc42–ACK GBD structure showing the contacts made by Leu-485^ACK^ and Leu-487^ACK^. *Coloring* is as in *A*. The magnesium ion in the structure of Cdc42 is shown as a van der Waals surface and *colored orange*. Where highlighted, oxygen atoms are *colored red* and nitrogen in *blue*.

I454A (mutating the first conserved residue of the CRIB motif) has the most dramatic effect of any single mutation tested, binding of this mutant being undetectable in direct SPAs, whereas the competition SPA gives a *K_d_* ∼1000-fold higher than wild type. Ile-454 is lodged in a hydrophobic pocket, making extensive and clearly important contacts with Leu-177, Ile-46, Gly-47, and Gly-48 of Cdc42 (Fig. 4*A*). S455A (mutating the second CRIB conserved residue) has a less dramatic, although still influential, effect on binding; contacts to Cdc42 seen in the structure are largely through the backbone at this position.

Pro-457 (CRIB consensus residue 3) is part of a kink in the ACK GBD that projects into a hydrophobic pocket (toward Phe-169^Cdc42^, shown in Fig. 4*B*), and mutation to alanine has an interesting effect on binding. P457A is one of the mutations that produced poor data in direct binding assays. The competition data show that binding affinity for P457A is reduced to around 4 μm (Table 2), indicating that this mutation has an ∼190-fold effect on overall affinity. The protein backbone of ACK bends tightly at Pro-457, and this residue likely stabilizes the whole binding region in the correct orientation for binding. In place of the proline, alanine should not disrupt the hydrophobic character of the region; however, the backbone of the region would be more flexible because proline fixes the φ angle, whereas alanine has more rotational freedom. Pro-457 thus stabilizes a section of the peptide into a relatively tight bend, which follows the topography of Cdc42. Binding to Cdc42 demands a loss of conformational entropy for the GBD (27); the ACK GBD is unstructured when unbound but must adopt a specific conformer to bind. Proline replacement with alanine would increase conformational entropy of the free GBD and therefore the energetic cost of binding (28), accounting for the dramatic lowering of the overall binding affinity observed.

Between Ser-461 and His-464 there is a region of the ACK GBD that forms a small section of antiparallel β-sheet with Ala-41 to Met-45 of Cdc42 upon complex formation. The presence of this intermolecular β-sheet is very common in small G protein–effector complexes (7) and indicates the likely existence of hydrogen bonds across the interface. Mutation of Ser-461, Phe-462, or His-464 disrupts complex formation significantly (with mutation of Ile-463 showing a more modest effect), suggesting important side-chain interactions, with S461A and F462A mutants causing particularly large decreases in binding affinity (∼200- and 170-fold, respectively).

Data from the Cdc42–ACK structure suggest that the side chain of Ser-461^ACK^ can form hydrogen bonds with Thr-43^Cdc42^ (Fig. 4*C*), whereas Phe-462^ACK^ is positioned over a hydrophobic patch on helix α1 of Cdc42 and is particularly close to Ile-21 and Leu-20. Our structure shows that the backbone of Phe-462 contacts Tyr-40^Cdc42^ and Val-43^Cdc42^, whereas its side chain contacts Leu-20^Cdc42^, Tyr-24^Cdc42^, and Val-42^Cdc42^ (Fig. 4*C*). As the backbone chain in this region of ACK interacts with the antiparallel β strand from residue 40 to 45 in Cdc42, forming the intermolecular β-sheet, it is possible that a large residue like Phe-462 is necessary to interact with the hydrophobic section of helix α1 while simultaneously allowing the backbone to make favorable contacts with the β-strand in Cdc42.

I463A^ACK^ shows a slightly reduced affinity for Cdc42 of around 3.5-fold. Mutation to alanine possibly disrupts the contacts formed with the γ-carbon of Thr-43^Cdc42^ and with Ala-41^Cdc42^. Val-42^Cdc42^ also lies close to Ile-463^ACK^, and this interaction is likely to be important, because the V42A mutation is very disruptive for ACK–Cdc42 binding (20). However, because this mutation has a relatively minor effect on binding here and a previous double mutant cycle study that we undertook concluded that the interaction between Val-42 and Ile-463 was not crucial to binding (27), the disruption due to V42A^Cdc42^ mutation is more likely to be due to interaction with other critical residues, for instance Phe-462 (see above).

The two invariant histidines in the CRIB region, His-464^ACK^ and His-467^ACK^, have been identified as vital to CRIB effector binding and have been mutated, either together or singly, to prevent CRIB protein interaction in many studies (29–32). The residues are predicted to form hydrogen bonds with Asp-38^Cdc42^, which are vital for CRIB–effector binding. These residues are not well defined in the Cdc42–ACK complex, but D38A^Cdc42^ causes a 67-fold reduction in ACK binding (8, 20). In competition assays, *K_d_* values for H464A and H467A were increased 125- and 89-fold, respectively (Table 2). This demonstrates that interactions with residues other than Asp-38^Cdc42^ are likely (*e.g.* His-464^ACK^ also makes contacts with Phe-37^Cdc42^ and Tyr-40^Cdc42^). Although the histidines contribute significant binding energy, they are less critical, individually, than all other CRIB consensus residues except Ser-455. We also tested a double H464A/H467A mutant because this combination has been used in the literature as a non-binding mutant; binding of H464A/H467A to Cdc42 was completely undetectable in both direct and competition SPAs. This indicates that the combination of both histidines is necessary for stabilizing the Cdc42–ACK complex and that their combined loss is fatal to binding. Studies requiring a non-Cdc42 binding mutant of ACK would therefore be best facilitated using a double histidine mutant or I454A.

### Residues significantly affecting Cdc42 binding at the N terminus of the ACK GBD

S450A and A451D both reduce the binding affinity for Cdc42 by ∼4-fold. Ser-450 lies close to the carboxyl group of Glu-181 in various conformations of the family of NMR structures, possibly supplying a polar interaction here. A451D is potentially sterically disruptive and alters the hydrophobic character of the area, so it is of little surprise that it increases the *K_d_*. Mutation of Leu-449 proved more significant, increasing the *K_d_* 11-fold. This residue forms significant contacts with Leu-174 (Fig. 4*E*). Mutation of Leu-174^Cdc42^ to alanine has been shown to increase the dissociation constant by 30-fold in previous work, which also reported that L449A decreases the affinity by 11-fold (20, 27). The hydrophobic interaction between these leucines is clearly important, in agreement with our previous study, although mutation of Leu-174^Cdc42^ is more disruptive, potentially due to its interactions with Ala-451^ACK^. Leu-449 contributes the most energy of any individual side chain outside the CRIB to the total binding energy of the complex at 1.43 kcal/mol, or 14.3% (as assessed in direct binding assays), indicating an important role for the N terminus of the ACK GBD.

### Energetic contributions at the C terminus of the ACK GBD

The W476A mutation in ACK gives rise to an ∼3-fold increase in affinity for Cdc42. This relatively minor change is likely due to the loss of a hydrophobic interaction with Phe-28^Cdc42^, which is 4 Å from Phe-476^ACK^ in the NMR structure (8).

Mutation of Asp-480 to alanine increases the *K_d_* of ACK for Cdc42 by ∼4-fold; this is likely due to the loss of hydrogen bonds between Asp-480^ACK^ and Ser-83^Cdc42^. The mutant R481A shows a more modest decrease in affinity of 2.4-fold to 107 nm. This residue adopts various conformations in the NMR family of structures but most likely forms contacts with Tyr-32^Cdc42^ and has the potential to form a hydrogen bond with the backbone oxygen of Phe-28^Cdc42^. However, the flexibility observed in the NMR structures suggests a degree of transience in these interactions.

The leucines at positions 485 and 487 in the ACK GBD have been postulated to interact with Leu-67 and Leu-70 of Cdc42 (8). Interestingly, the mutation L485A had no effect on Cdc42 binding, whereas L487A reduced the affinity 7.5-fold. From examination of the NMR structure, Leu-485^ACK^ forms contacts with Leu-70^Cdc42^ but is also solvent-exposed, whereas Leu-487^ACK^ makes contacts with Thr-58–Gly-60^Cdc42^ and is encased by Cdc42 residues in the complex structure. Potentially, alanine can replace the hydrophobic interactions of Leu-485^ACK^ sufficiently so as not to disrupt the complex, whereas Leu-487^ACK^, embedded in the complex, is more important and may well clamp the C-terminal end of the ACK GBD to Cdc42 (Fig. 4*F*).

Our data demonstrate that the majority of the energetically important residues for the interaction between Cdc42 and ACK are located toward the N terminus of the ACK GBD, so we postulated that a C-terminally truncated form of the ACK GBD might still retain reasonable affinity for Cdc42. Therefore, the association of a truncated section of the ACK GBD terminating at Pro-472 (named Δ17) with Cdc42 was investigated. This peptide retains the full CRIB consensus motif and the majority of the energetically important residues identified in this study. Although binding of this peptide could not be determined by direct SPAs (Table 1), it did compete with wild-type ACK, albeit with a significantly reduced affinity of 4.8 μm (Table 2). The removal of these 17 C-terminal residues had, however, only an effect of similar magnitude to mutations of certain single residues (*e.g.* S461A) and proved less disruptive than either the H464A/H467A double mutant or I454A. Overall, the 17 residues that comprise this part of the GBD provide ∼3.2 kcal/mol to the total binding energy (∼31%). It is possible, therefore, that a shorter peptide based on the N-terminal sequence might still bind tightly enough to Cdc42 to disrupt the Cdc42 interaction with ACK and other CRIB effector proteins via this crucial region.

### Energetically neutral residues

ACK mutations Q452A, Q459A, D469A, S470A, and L485A produced no changes in Cdc42 binding despite being identified as being either near energetic hot spots on Cdc42 or flagged up by computational predictions. Because these residues do not seem to contribute thermodynamically to the Cdc42–ACK binding interface, they represent an important cohort of residues that could be changed to improve binding.

### Kinetics of the interaction between ACK and Cdc42

**Figure 5.**
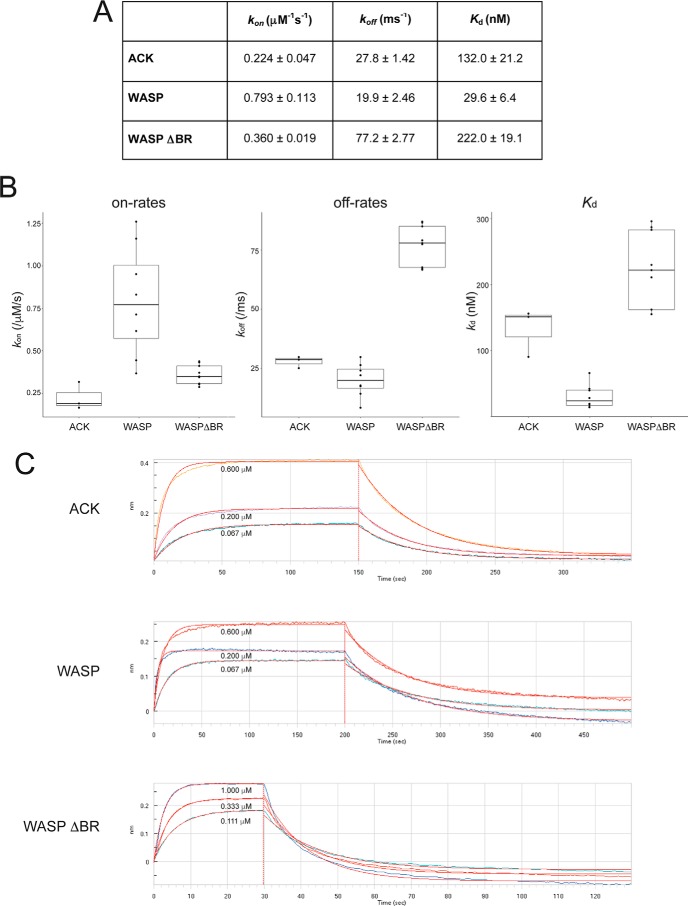
**Binding kinetics for ACK, WASP, and WASP ΔBR measured by bio-layer inferometry.** GST fusion proteins were loaded onto anti-GST sensors and dipped alternately into a concentration range of Cdc42·GMPPNP (of at least three different concentrations) and buffer to measure on- and off-rates. *A*, the *table* shows the mean values for *k*_on_, *k*_off_, and *K_d_* with S.E., where *n* = 3–9 independent experiments. *B*, the same data as summarized in *A* plotted as *box plots* (analysis performed in R). *C*, raw data from representative experiments. *Error bars*, S.E.

The WASP–Cdc42 interaction has been demonstrated to occur using a dock and coalesce mechanism driven by electrostatics (18, 19). We were interested, therefore, in analyzing the kinetics of the ACK–Cdc42 interaction to see whether a similar mechanism of binding was utilized. We analyzed the ACK–Cdc42 interaction using bio-layer inferometry (BLI), and the results are shown in Fig. 5. Summaries of the kinetic data are shown in *A* and *B*, with representative raw data in *C*.

The overall affinity of ACK–Cdc42 was calculated to be ∼130 nm, in good agreement with the SPA data. The on-rate was 0.224 μm^−1^ s^−1^, whereas the off-rate was ∼28 ms^−1^. For comparison, we also undertook a similar analysis for WASP–Cdc42 and a variant of WASP lacking the electrostatic steering region (ΔBR). The WASP–Cdc42 affinity was calculated to be ∼30 nm, tighter than the ACK–Cdc42 complex and in agreement with previous data (20). The higher affinity of WASP for Cdc42 is driven by a 3-fold increase in its *k*_on_ to 0.793 μm^−1^ s^−1^ compared with that of ACK, in conjunction with a similar off-rate of ∼20 ms^−1^, as might be expected for a CRIB protein with an electrostatic steering region. The WASP ΔBR variant (with all six basic residues at the N terminus of the WASP GBD mutated to alanine) showed an affinity of ∼220 nm, comprising an on-rate of 0.360 μm^−1^ s^−1^ and an off-rate of 77 ms^−1^. The on-rate of WASP ΔBR is half that of WASP and closer to that of ACK, as would be predicted from the loss of the steering residues. Interestingly, however, the lower affinity of the mutant is also due to a much faster off-rate (∼4-fold higher) than WASP.

To identify the residues involved in the docking phase of the ACK–Cdc42 interaction, we attempted to analyze our ACK mutant variants by BLI, rationalizing that mutation of these residues would result in a decrease in ACK *k*_on_. However, because the affinity of these ACK mutant variants was comparably low, high concentrations of protein would be required to obtain useable data. Unfortunately, the higher concentrations of protein led to increased levels of nonspecific binding, and the BLI data became unreliable, preventing further analysis.

## Discussion

**Figure 6.**
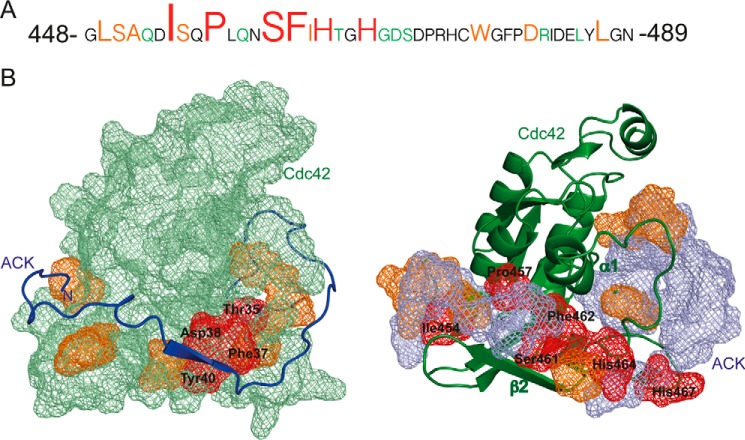
A, *schematic representation* of the ACK GBD with residues scaled by size relative to their contribution to Cdc42 binding affinity. Residues whose mutation causes < 3-fold increase in *K_d_* are *colored green*; those whose mutation causes a 3–12-fold increase are *colored orange*; and those whose mutation causes a > 12-fold increase are shown in *red. B*, structural and thermodynamic details of the Cdc42–ACK complex (PDB entry 1CF4). *Left*, the van der Waals surface of Cdc42 as a *green mesh*; residues whose mutation causes a 3–12-fold increase in *K_d_* are *colored orange*, and those leading to a >12-fold increase are *colored red* and *labeled*. ACK residues 448–489 are shown as a *schematic* in *blue*. The N terminus of ACK is *labeled. Right*, structure of Cdc42 as a *schematic* in *green*. The van der Waals surface of ACK residues 448–489 is shown as a *blue mesh*, residues whose mutation causes a 3–12-fold increase in *K_d_* are *colored orange*, and those leading to a > 12-fold increase are *colored red* and *labeled*. β-Strand 2 and α-helix 1 of Cdc42 are *labeled* accordingly.

**Figure 7.**
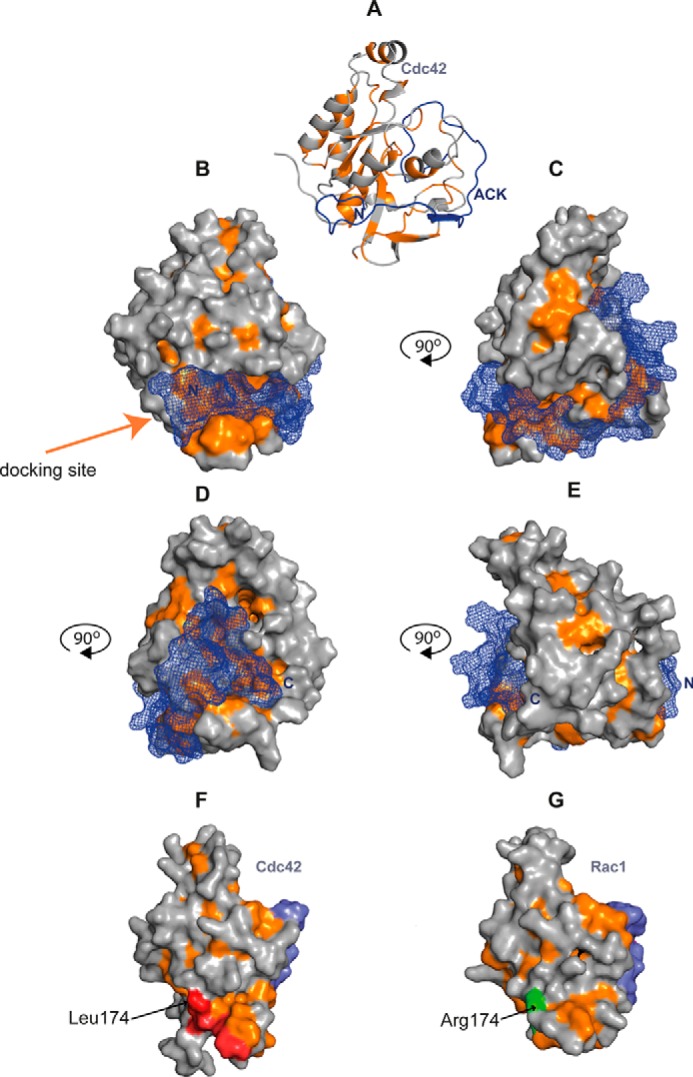
*A*, *schematic* representation of the Cdc42–ACK structure (PDB entry 1CF4). Cdc42 is shown in *gray*, with hydrophobic residues (Ala, Gly, Val, Ile, Leu, Phe, and Met) *colored orange*. ACK is shown in *blue*, and its N terminus is *labeled. B*, van der Waals surface of Cdc42–ACK structure shown in the same orientation as in *A*. The van der Waals surface of Cdc42 is *solid*, whereas ACK residues 448–489 are shown as a *blue mesh. C*, as in *B*, but with the Cdc42–ACK structure rotated 90° around the vertical, anti-clockwise. *D*, as in *B*, but with the Cdc42–ACK structure rotated 180° around the vertical, anti-clockwise. *E*, as in *B*, but with the Cdc42–ACK structure rotated 270° around the vertical, anti-clockwise. *F*, van der Waals surface of Cdc42·GMPPCP shown in *gray*, with hydrophobic residues (Ala, Gly, Val, Ile, Leu, Phe, and Met) *colored orange*. Leu-174, Leu-177, Ile-46, Gly-47, and Gly-48 are shown in *red* (PDB entry 2QRZ). Switch I residues are *colored blue. G*, van der Waals surface of Rac1 Q61L·GMPPNP shown in *gray*, with hydrophobic residues (Ala, Gly, Val, Ile, Leu, Phe, and Met) *colored orange*. Arg-174 is shown in *green* (PDB entry 2GZL). Switch I residues are *colored blue*.

The data presented here indicate several residues that are key to Cdc42–ACK binding, and these are centered on the CRIB region. An overall summary of the relative contribution that each ACK residue of the GBD makes to the Cdc42–ACK complex is shown in Fig. 6*A*. How these energetic contributions map onto the structure of the complex is shown in Fig. 6*B*, along with the complementary contributions of the Cdc42 residues. Overall, most binding energy resides in the center of the ACK GBD, concentrated in CRIB consensus residues. The region is anchored to Cdc42 at each end by hydrophobic interactions. We therefore envisage that initial docking is mediated by the N-terminal region of the ACK GBD in a manner akin to the electrostatic steering or basic region docking demonstrated for Cdc42-WASP and postulated for Cdc42-PAK (18, 19) but differing significantly by being driven by hydrophobic contacts. The patch of residues on ACK comprising Leu-449, Ser-450, and Ala-451, contacts Cdc42 via residues in helix α5 and the C terminus of the protein (*e.g.* Glu-181^Cdc42^). This region is not subject to the rearrangements experienced by the switches and would provide a relatively stable platform to mediate the initial docking phase of complex formation and therefore facilitate molecular recognition for the two partners. This hydrophobic patch interaction would be further extended by Ile-454^ACK^, which contacts Leu-177, Ile-46, Gly-47, and Gly-48^Cdc42^, all of which are structurally stable, nucleotide-independent residues. The hydrophobic potential of the surface of Cdc42 is shown in Fig. 7. An intense concentration of hydrophobic potential is seen on Cdc42, complementing the N-terminal hydrophobic residues of ACK (Fig. 7, *A* and *B*). Although other regions of hydrophobicity are seen on Cdc42 (Fig. 7, *C–E*), none are comparable with the density seen at this pocket, giving it plausible docking potential. This hydrophobic potential is also seen in free Cdc42 in the active form (Fig. 7*F*) and so would be available to form an encounter complex. Interestingly, the same intensity of hydrophobic potential is absent from the surface of Rac1 (to which ACK does not bind), where the presence of Arg-174 at least partially disrupts the potential (Fig. 7*G*). Leu-174^Cdc42^ was identified previously as a specificity determinant for ACK binding (20).

It is well accepted that small G proteins like Cdc42 are only able to bind effectors when bound to GTP. However, it appears that the native structure of Cdc42 changes little on nucleotide exchange and is possibly only finally stabilized in the active conformation on effector binding (5). The mechanism suggested to underpin this involves the effector initially binding loosely, creating a favorable hydrophobic cleft around Phe-37^Cdc42^ and Val-36^Cdc42^ (in our previous study, V36A was found to decrease binding affinity > 10-fold (20)). The resulting movement of switch I is then stabilized by Thr-35^Cdc42^ coordination to the γ-phosphate of GTP and to Mg^2+^. When GDP is bound, this cannot occur, and the effector complex is transitory; likewise, without the effector protein, the active conformation is also transitory (5).

In this model, ACK residues in the vicinity of Phe-37^Cdc42^ and Thr-35^Cdc42^ would be of critical importance. The section of ACK between Ser-461 and His-467 contains a concentration of residues that our data indicate are vital for binding (Fig. 6). The most important appear to allow the GBD to interact with two distinct surfaces of Cdc42, strand β2 (forming the intermolecular β-sheet) and helix α1 of Cdc42, inserting between these to stabilize the quaternary complex (Fig. 6*B*). His-467^ACK^, which faces away from Val-36^Cdc42^ but is close enough to Asp-63^Cdc42^ for a polar interaction (Fig. 4*D*), contributes a hydrogen bond (predicted by our structural data). It is possible that this association provides a key point of contact with switch II, allowing formation of the required hydrophobic cleft for stable effector association. His-464^ACK^ contacts Phe-37^Cdc42^ and so would also contribute to stabilizing switch I. After initial docking, it seems that the conserved histidines of the CRIB, assisted by other nearby residues, help to grasp switches I and II and create an environment where Thr-35^Cdc42^ can interact both with the γ-phosphate of GTP and Mg^2+^ to create a stable complex. Key mutations in the central CRIB region would destabilize this coalescence part of complex formation, as observed in the poor binding affinities of CRIB mutants.

Thus, ACK could bind Cdc42 in an initial, transient manner mediated by the N-terminal region of the GBD constituting the docking or encounter complex (Fig. 7*B*). Residues such as Ile-454^ACK^, whose mutation disrupts binding profoundly, are likely to be involved in docking complex formation via interaction with stable, structured regions of Cdc42. These stabilize the initial encounter complex by forming bracing interactions with less flexible Cdc42 residues. Subsequent interaction, probably by contacts to switch I, would then initiate coalescence of the second section of the complex, stabilizing the active conformations of the switch regions of Cdc42 and locking the ACK GBD in its binding configuration. Mutation of certain critical side chains (*e.g.* Ser-461^ACK^ or Phe-462^ACK^), however, would hinder the stabilization of the bound structure in a similar manner to the absence of the γ-phosphate in the GDP-bound form. Thus, unlike many IDP interactions, where the target protein is structured, in the Cdc42–ACK complex, the disordered structure of both the effector and the target binding interface are together stabilized into the active signal-transducing conformation by a reciprocal rigidifying mechanism.

In light of this new data set, we can match the effects of mutation in the ACK GBD to the other side of the binding interface. Two mutations in Cdc42 that had a significant impact on ACK binding were F37A and D38A. These residues lie close to the CRIB region of ACK in the structure, and the histidines at positions 464 and 467 have been identified as forming hydrogen bonds with Asp-38 in several CRIB–Cdc42 complexes, as discussed earlier. Conserved CRIB residues, such as His-467, His-464, and Phe-462 of ACK, which surround residues 37–40 on Cdc42, have been shown in this study to be important for stabilizing the ACK–Cdc42 complex and so match the mutation data found previously.

F56W^Cdc42^ was previously suggested to be a mutation thought to disrupt the topography of Cdc42 that is appropriate for ACK binding, because it does not form direct contacts with ACK but does alter the complex binding affinity (21). Phe-56^Cdc42^ packs behind Phe-37^Cdc42^ and Asp-38^Cdc42^, which have been implicated in binding specificity. This is consistent also with a model where effector binding stabilizes switch I, sandwiching it between Cdc42 and the GBD. F567W disrupts the packing behind switch I and prevents the stable conformation forming that allows secure effector binding.

### Comparing Cdc42 effector proteins

ACK and WASP are both Cdc42-specific CRIB effectors, whereas PAK1 is not (being able to also bind Rac1), and the CRIB regions bind to Cdc42 in a similar manner. Comparison of the structures, together with thermodynamic data, is informative in deciding which interactions may be Cdc42-specific.

N-terminal to the CRIB, the hydrophobic interactions around Leu-449 appear crucial for ACK binding to Cdc42. There is a degree of conservation at least in the character of the residue at the position analogous to Leu-449^ACK^ among other Cdc42-specific effectors, as seen in Fig. 1*B*. In the Cdc42-WASP structure, the N-terminal region of the GBD is in contact with a similar region of Cdc42 to the ACK-binding region. Ile-273 (the equivalent residue) in WASP occupies a position similar to that of Leu-449 of ACK, close to Leu-174^Cdc42^ and even closer to Leu-177^Cdc42^. This region differs in the PAKs and lies outside of the region necessary for high-affinity PAK1 binding to Cdc42 (33); indeed, the L174A mutation in Cdc42 has a minimal effect on PAK1 binding. Because PAK1 is a more promiscuous binder, the Leu-174/Leu-449 interaction appears to be an important determinant for specific Cdc42 binding. Another Cdc42-specific effector, Cdc42SE2 (or SPEC2) (34), has an isoleucine at this position, complementing the theory that a bulky hydrophobic residue here is key for a CRIB protein that is specific for Cdc42. In Rac1 and RhoA, Leu-174^Cdc42^ is replaced by an arginine, which would clash with the bulky hydrophobic Leu/Ile in the Cdc42-specific effectors, in contrast to the stabilizing influence provided by Leu-174 in Cdc42 (Fig. 4*E*).

Pro-457 and Ser-461, which are crucial for ACK binding, are also present in WASP; however, two of the intervening residues are missing. Ser-242^WASP^, like Ser-461^ACK^, lies very close to the backbone hydroxyl of Thr-43^Cdc42^, potentially forming a similarly important hydrogen bond. However, Pro-241^WASP^ (equivalent to Pro-457^ACK^) is now adjacent to Ser-242^WASP^, so there is likely some difference in this residue's contribution to binding in WASP–Cdc42. With the ACK sequence being slightly longer than WASP here, the kink that is stabilized in the ACK peptide chain around Pro-457 may be important in allowing the ACK chain to occupy the optimal position on the surface of Cdc42; indeed, the proline appears in a similar position but buried deeper in the ACK–Cdc42 structure than that of WASP–Cdc42, facilitated by the extra residues. Overall, the GBD topography between effectors is similar between the conserved Ile-454 and His-467 (ACK numbering), with the caveat that ACK is able to insert deeper into the Cdc42 structure between helix 1 and the C-terminal end of switch II due to its extended sequence in this region. This region is important for ACK binding affinity, but possibly less so for distinguishing between different CRIB effectors (ACK relies on Val-42, whereas WASP and PAK1 can still bind without it; Rac1 has the smaller Ala at this position, which may discourage ACK binding). It is notable from our kinetic data that, once the basic region residues of WASP have been removed, the off-rate for WASP is shown to be faster than that for ACK (Fig. 5). This is possibly due to the more superficial interactions between Cdc42 and WASP dictated by this region.

Val-42^Cdc42^ is an interesting discriminatory residue in that its mutation affects ACK but not WASP or PAK1 (20). This residue is proximal to Phe-462 and Ile-463 in ACK, both of which could interact with the hydrophobic side chain; however, because only the phenylalanine is conserved across CRIB effectors, the discrimination is likely to be due to Ile-463. I463A^ACK^, however, only reduces the affinity 3.5-fold, which cannot explain the nearly 20-fold decrease generated by V42A^Cdc42^. As the comparison of the structures has shown, ACK inserts deeper into Cdc42, whereas WASP simply lies over the C terminus of switch II, which could allow Phe-462^ACK^ improved access to Val-42^Cdc42^ and mean that the hydrophobic interaction here is more important than for other Cdc42 effectors.

Toward the C terminus of the GBD, fewer residues in ACK appear important for the binding affinity, although, since they overlie switch I, they likely help pin the entire active conformation of Cdc42 into place. In agreement with the overall character of the Cdc42–ACK binding interface, both ends are in fact tied down by important hydrophobic interactions (Figs. 6 and 7).

### Biological implications

The differences between the WASP and ACK GBDs and their respective binding mechanisms with Cdc42 likely reflect evolutionary responses to the different roles of the GBDs in the complete effector proteins. The negative regulation of WASP (and indeed PAK1 (35)) by intramolecular interactions between the GBD and functional domains, which are relieved by the binding of Cdc42 to the GBD, is well documented (36). The regulation of ACK is less well understood, and despite significant attempts to unravel interactions governing its activity, results continue to be contradictory. Analysis is hampered by the fact that ACK function is often measured in terms of its kinase activity, when ACK clearly has kinase-independent cellular functions. Two levels of intramolecular regulatory interactions in ACK do seem likely, however: the inhibitory interaction between the MHR and the kinase domain (37) and dimerization (probably via the SAM domain) that activates kinase activity (38). However, despite ACK being initially identified as an effector for Cdc42, it now seems unlikely that binding of Cdc42 directly stimulates the kinase activity of ACK. Any increase in activity seen in cells is more likely due to subcellular localization effects. It therefore seems evident, at least from information currently available, that the ACK GBD is freely available to associate with Cdc42. These differences are reflected in the relative affinities of the isolated GBDs and the full-length proteins for Cdc42. In WASP, the GBD binds Cdc42 significantly tighter (∼100 times) than the full-length protein (9, 39), whereas for ACK, the GBD and longer constructs bind with similar affinity (11). The corollary of this scenario is that WASP needs to form an encounter complex for Cdc42 that utilizes residues outside the GBD, which are available for interaction even in its autoinhibited conformation. Because the ACK GBD is not involved in intramolecular interactions, it is free to steer recognition of Cdc42 itself. This is reflected in the lower *K_a_* measured for ACK–Cdc42 in comparison with WASP–Cdc42.

One of the driving forces for this analysis of the Cd42–ACK binding interface was to generate data to inform rational therapeutic design. The full ACK GBD used to solve the Cdc42–ACK structure has been shown to reverse Ras-driven transformation when introduced into cell lines (14). The data presented here demonstrate that the majority of the binding energy of the ACK GBD resides between Leu-449 and His-467; thus, a peptide containing at least these residues would provide an excellent starting point for a therapeutic. These data also define the target region of chemical space for fragment-based drug design. Furthermore, by highlighting residues that do not contribute to the Cdc42–ACK interface, important points are identified at which a peptide could be engineered with increased affinity, by substituting amino acids that introduce further favorable contacts with Cdc42. It is also notable that ACK lacks the electrostatic steering mechanism employed by WASP and probably PAK1. It is possible, therefore, that the addition of a positively charged motif to the N terminus of ACK could further increase binding of this effector to Cdc42, by exploiting the negatively charged patch provided by Glu-49 and Glu-178 on Cdc42 and increasing the on-rate of ACK to WASP levels (19).

### Conclusion

A model of Cdc42–effector binding involving a dock and coalesce mechanism initiated by electrostatic steering is evolving, where the interaction with the effector protein stabilizes switch I in the active conformation, bringing the effector GBD and the flexible parts of Cdc42 into a stable quaternary structure (5, 18). Binding data presented here for Cdc42–ACK correlate with this model. Energetically important residues in ACK are primarily concentrated in the N terminus and CRIB consensus of the GBD (Fig. 6). Like WASP, ACK may also bind initially via its N-terminal energetic hot spot, where the complementary Cdc42 structure is more rigid regardless of activation state and would therefore provide a good docking subsite. In the case of WASP, and possibly PAK1, this is assisted by a polylysine-directed electrostatic steering mechanism, with the steering section not an energetically important part of the final complex. In ACK, the encounter would be directed by hydrophobic interactions that also remain as part of the final complex (Fig. 7). Following N-terminal docking, the remainder of the ACK GBD can wrap around Cdc42, stabilizing switches I and II in their active conformation and leading to a final structured, stable, quaternary complex. Several consensus CRIB residues toward the center of the GBD are vital in the stability of this final structure, inserting between switch I and helix α1 of Cdc42. The similarity of these residues between CRIB effectors implies that they are not key to the specificity of ACK for Cdc42. The C-terminal region of the GBD contributes to the overall binding affinity through several dispersed residues.

The data reported here support a dock and coalesce mechanism for ACK and suggest that this could describe a general mechanism whereby these unstructured GBDs interact with their cognate small G protein in common with many intrinsically disordered proteins. Cdc42–ACK, however, represents a new category of this type of interaction being instigated by hydrophobic contacts. Initial docking is mediated by a hydrophobic patch on ACK, which may perform a role similar to that of the electrostatic steering region of other CRIB effectors. This is followed by the coalesce phase, mediated by the CRIB motif, which orientates Phe-37^Cdc42^, allowing Cdc42·GTP to lock into the active “state 2” conformation. The rest of the binding interface then zips up, and residues on Cdc42 clip ACK into place to form the final stable complex.

## Experimental procedures

### Protein expression constructs

Proteins were expressed in pGEX-2T vectors (Amersham Biosciences) as GST fusion proteins. The constructs expressing ACK(448–489) (8) and Cdc42 Δ7 Q61L (21) have been described previously.

### Mutagenesis

Residues were mutated to alanine, with the exception of Ala-451, which was mutated to aspartate. Site-directed mutagenesis on ACK(448–489) was performed using the QuikChange Lightning multisite-directed mutagenesis kit (Agilent), and mutations were confirmed by sequencing (Department of Biochemistry DNA Sequencing Facility, University of Cambridge).

### Recombinant protein production

*Escherichia coli* BL21 cells were used to express recombinant proteins. Stationary cultures were diluted in fresh 2TY growth medium (1:10) and grown to *A*_600_ 0.7–0.9 at 37 °C, whereupon they were induced with isopropyl-β-d-1-thiogalactopyranoside (0.1 mm) for 5 h. Proteins were affinity-purified on glutathione-agarose resin (Sigma-Aldrich). Cdc42 and ACK mutants used for competition assays were cleaved from GST using thrombin (Novagen), whereas GST–ACK proteins for direct binding assays were eluted from the resin in 10 mm reduced glutathione (in 50 mm Tris–HCl, 150 mm NaCl, pH 7.5). Cdc42 and cleaved ACK mutants were further purified by gel filtration (S75 16/60 and S30 16/60, respectively; GE Healthcare), whereas GST–ACK mutants were purified using ion exchange (Resource Q 1 ml; GE Healthcare). Protein concentrations were measured by *A*_280_ using calculated extinction coefficients.

### Nucleotide exchange

Cdc42 was labeled with [^3^H]GTP as follows. [^3^H]GTP (PerkinElmer Life Sciences) was dried by centrifugal evaporation, and 0.7 mg of Cdc42, 15 nm phosphoenol pyruvate, 6 units pyruvate kinase (Sigma-Aldrich), 15 mm KCl, and 0.36 m (NH_4_)_2_SO_4_ were added in 140 μl of buffer (10 mm Tris–HCl, 150 mm NaCl, 1 mm DTT, pH 7.5). The mix was incubated at 37 °C for 3 h, after which 10 mm MgCl_2_ was added to quench the reaction. Unbound nucleotide was removed with Sephadex G25 spin columns (GE Healthcare).

### SPAs

#### 

##### Direct binding

Affinities of ACK proteins for Cdc42 were measured using SPA. 20 nm concentrations of individual ACK mutants were immobilized on protein A SPA fluoromicrospheres via an anti-GST antibody (Sigma-Aldrich). The equilibrium binding constants (*K_d_*) of the effector–G protein interaction were determined by monitoring the SPA signal in the presence of varying concentrations of [^3^H]GTP–Cdc42, as described previously (21). Binding of Cdc42 to the effector domain brings the radiolabeled nucleotide into proximity with the scintillant, allowing a signal to be generated. For each ACK mutant, a negative control was performed in the absence of effector, which resulted in a linear increase in background SPA counts. This data set was then subtracted from the data points obtained in the presence of effector, and the adjusted values were plotted as a function of increasing concentration of Cdc42. For each affinity determination, data points were obtained for at least eight different G protein concentrations. Binding curves were fitted using a direct binding isotherm (33) to obtain *K_d_* values and their S.E. values for the G protein–effector interactions.

##### Competition binding

In cases where binding of Cdc42 to ACK mutants was relatively weak (reducing the quality of the binding curve fit), the mutants were used to compete with the interaction between WT ACK GBD and Cdc42. 30 nm WT ACK was immobilized on protein A SPA fluoromicrospheres via an anti-GST antibody (Sigma-Aldrich). 30 nm Cdc42 was added, and the effect of competition was measured by measuring the signal in the presence of increasing concentrations of (GST-cleaved) ACK mutant peptide. SPA counts were measured at each concentration and fitted to the appropriate binding isotherm as described (27).

### Bio-layer inferometry

The interactions between Cdc42 and GST fusion constructs of ACK, WASP, and a basic region mutant of WASP were studied using bio-layer inferometry on an Octet Red system (ForteBio). Experiments were performed in 50 mm Tris–HCl, pH 7.5, 150 mm NaCl, 1 mm MgCl_2_, 0.02% Tween 20, 0.1% BSA, and 0.05% sodium azide at 25 °C. Anti-GST sensors were loaded with GST fusion proteins at concentrations of 10 μg/ml, washed to return to baseline, and then dipped into a dilution range of Cdc42·GMPPNP to measure association. Dissociation was measured by dipping sensors into buffer after the association step. To minimize the effects of nonspecific binding, binding data were collected for sensors loaded with GST alone, and this was subtracted from the experimental traces. Sensors were regenerated in 10 mm glycine, pH 2, for reuse. Analysis was undertaken using the Octet Red analysis software.

## Author contributions

G. J. N. T. performed the experiments. G. J. N. T., H. R. M., R. N. C., and D. O. analyzed the data and interpreted the experimental results. G. J. N. T., H. R. M., R. N. C., and D. O. designed the experiments and wrote the paper.

## Footnotes

3 The abbreviations used are:

GMPPCP: guanylyl β,γ-methylenediphosphonate
GMPPNP: guanosine 5′-β,γ-imidotriphosphate
BLI: bio-layer inferometry
GBD: G protein-binding domain
SPA: scintillation proximity assay
CRIB: Cdc42/Rac interactive binding
BR: basic region
ACK: activated Cdc42 kinase
WASP: Wiskott-Aldrich syndrome protein
PAK: p21-activated kinase
IDP: intrinsically disordered protein
IDR: intrinsically disordered region
PDB: Protein Data Bank.
